# Evaluating the positional uncertainty of intrafraction, adjacent fields, and daily setup with the BrainLAB ExacTrac system in patients who are receiving craniospinal irradiation

**DOI:** 10.1002/acm2.12909

**Published:** 2020-06-03

**Authors:** Xiaojuan Duan, Yibing Zhou, Hongya Dai, Lirong Zhao, Jindong Qian, Dingqiang Yang, Liwei Zhang, Can Luo, Guanghui Li

**Affiliations:** ^1^ Institute of Cancer Research Xinqiao Hospital ARMY Medical University Chongqing China

**Keywords:** CSI, dose homogeneity, ExacTrac system, intrafraction error, junctions, setup

## Abstract

**Purpose:**

To investigate the daily setup, interfraction motion, variability in the junction areas, and dosimetric effect in craniospinal irradiation (CSI) patients.

**Methods:**

Fifteen CSI patients who had undergone split‐field IMRT were followed in the study. Previous, middle, and posttreatment, each target volume position was evaluated using the ExacTrac system. Interfraction and intrafraction motions, the margin of the junction in adjacent targets volumes, and the dosimetric effect of the longitudinal residual error were analyzed.

**Results:**

The lowest attainment rate within the tolerance of the initial setup error was 66.79% in six directions. The values of the initial error were within 15 mm (SD 4.5 mm) in the translation direction and 5° (SD 1.3°) in the rotation direction after the transposition of the treatment isocenter. With the guidance of the ExacTrac system, the interfraction and intrafraction residual errors were almost within the tolerance after correction, the margin of CTV‐to‐PCTV was in the range of target expansion criteria. The residual longitudinal errors resulted in only slight changes in the mean doses of PGTV and PCTV, while the maximum dose of the spinal cord increased by 16.1%. The patients did not exhibit any side‐effects by the overall treatment during the follow‐up period.

**Conclusions:**

Position correction is necessary after setup and the transposition of the treatment isocenter. Intra‐fraction motion in the lateral direction should be monitored throughout treatment. The position errors in junction areas are almost within the tolerance after correction. The patients did not exhibit any side‐effects by the overall treatment.

## 1 INTRODUCTION

Radiotherapy (RT) or adjuvant RT following surgery is the current standard treatment for patients with an intracranial germinoma and medulloblastoma, which has dramatically increased the 5‐year survival rate; Craniospinal irradiation therapy (CSI) with linac is used as a primary treatment.^1^, ^2^ But the superlong target volume poses some technical challenges include beam matching, junctions, and gaps of fields that lead to dose heterogeneity in the junction area.^3^ A technique for overcoming these challenges is to shift the field boundaries weekly.^4^ Other techniques include extending the source‐to‐skin distance,^5^ the interactive movement of the couch,^6^ field‐in‐field,^7^ a linear ramp‐like dose profile,^8^ jagged‐junction IMRT^9^, and split‐field IMRT (sfIMRT),^3^, ^10^ and so on. The technique of sfIMRT with linac has become a major strategy for optimizing the whole craniospinal target volume simultaneously that offers several advantages, such as easier formulation of the radiotherapy plan, easier setup, reduced delivery time, more homogeneous dose, and superior sparing of organs at risk.^11^

The ExacTrac system (BrainLAB AG, Feldkirchen, Germany) is a patient positioning system consisting of a radiographic kV x‐ray imaging system for verifying patient position and a six degree‐of‐freedom (6D) robotic couch for correcting patient position in six‐dimensional directions.^12^, ^13^ ExacTrac offers several clinical benefits including faster patient alignment using the 6D robotic couch, the ability to monitor patient motion, and a reduction in image‐based radiation delivered to the patient,^14^ but it cannot provide much information due to a limited view of projections. In contrast, CBCT (cone‐beam CT) is favored because it offers a three‐dimensional view with better visualized anatomical structures and soft tissues than two‐dimension (2D) imaging options. However, its applications are limited by relatively long image acquisition time, relatively high radiation to the patient, and other technical limitations.^15^ An ideal IGRT method will minimize the dose without compromising the image guidance accuracy to prevent long‐term side effects by reducing the integral dose is highly important in pediatrics due to the long life expectancy of the patients. The accuracy of the ExacTrac system is reported, and the ExacTrac represents an alternative to CBCT for CSI.^12^, ^13^ Experiments have been done for CSI in our previous study,^16^ which prove that the accuracy of the ExacTrac system is consistent with the CBCT. The quality assurance of the infrared and the x‐ray system is done on a weekly and daily frequency, respectively. The weekly check includes the radiation isocenter defined by a Winston–Lutz test, the isocenter calibration aligning the couch top with the linac isocenter, and the x‐ray calibration calibrating with an ET (ExacTrac) X‐ray Calibration Phantom. The daily check verifies the ExacTrac isocenter and x‐ray calibration with a tungsten sphere located in the center of the ET Isocenter Phantom.

With the introduction of image guidance, positional variation can be measured and corrected in protocols.^17^ In this study, the technique of sfIMRT with linac and the ExacTrac system were used to observe daily setup, intrafraction motion, and positional errors on the junctions. Besides, despite great effort to achieve precise repositioning and immobilization of patients and image guidance, radiation delivery uncertainties still exist due to junctions, residual setup errors, and intrafractional involuntary variations during verification procedure. Thus, the quantitative dosimetric effects of positional uncertainties also need to be very well understood to ensure the delivery of high‐quality treatment to the CSI.

## 2 MATERIALS AND METHODS

### 2.A Patients

We report the outcomes of 15 unselected patients who were treated in our department between Oct. 2016 and Jan. 2018 with pathological detection, of whom 13 patients had germinomas, and two had medulloblastomas. The median age for these patients was 20 years (range 11–32 yr). All patients underwent tumor resection or biopsy. The pertinent information was tabulated (Table I).

**Table I:** Patient characteristics, prescribed radiation dose, and fraction size.

Patient	Histological diagnosis	Tumour location	Sex	Age(y)	Volume of PCTV(cm^3^)	Length of PCTV(cm)	No. of fractions	Prescribed dose of PGTV&PCTV(Gy)
1	germinoma	saddle area	F	11	2034.66	57.5	20	40 & 36
2	germinoma	pineal, hypophysis	M	19	2067.38	79.5	20	40 & 36
3	germinoma	saddle area, pineal	F	17	1999.46	73.5	20	45 & 36
4	germinoma	pineal	M	32	2410.32	80.4	20	45 & 36
5	germinoma	saddle area	M	14	2389.16	75.0	20	50 & 36
6	medulloblastoma	fourth ventricle	M	23	2163.29	80.0	20	52 & 36
7	germinoma	pineal	M	16	2628.54	83.0	20	44 & 36
8	germinoma	pineal	M	21	2431.34	87.0	20	44 & 36
9	germinoma	pineal	M	22	2103.67	82.0	20	45 & 36
10	germinoma	pineal, saddle area	F	20	1965.67	67.0	20	45 & 36
11	germinoma	pineal, saddle area	M	22	2047.76	78.5	20	45 & 36
12	medulloblastoma	opisthencephalon	F	12	2135.85	61.2	24	54 & 36
13	germinoma	saddle area	F	22	2426.96	74.4	20	45 & 36
14	germinoma	saddle area	M	26	2165.92	76.0	20	44 & 36
15	germinoma	saddle area	M	18	2146.20	75.0	22	50 & 36

Abbreviations: F, female; M, male.

### 2.B . CT simulation and treatment planning

The patients were immobilized in a head–neck–shoulder thermoplastic mask with their arms resting at their sides, while a tattoo line along the longitudinal axis of the body was drawn to facilitate setup. The GTV (Gross Tumor Volume) of the primary tumor and metastatic lesions was determined, and the CTV (Clinical Target Volume) included the whole brain, spinal cord, and terminal cisternae. The PGTV (Planning Gross Target Volume)/ PCTV (Planning Clinical Target Volume) was formed by adding a margin of 3 ~ 5 mm to the GTV/CTV. The following median were recorded: volume of PCTV 2146.2 cm^3^ (1965.67–2628.54 cm^3^), length of PCTV 76 cm (57.5–87 cm), fraction 20 (20–24), prescribed dose of PGTV 45 Gy (40–54 Gy). The detailed information was tabulated (Table 1). All planning target volumes were optimized in synchrony using a commercial TPS (Eclipse version 8.9; Varian Medical Systems, Palo Alto, CA). Colinear isocenters (iso1, iso2, and iso3) were placed in sequence. The iso1 was for cranial PCTV, iso2 was for the upper spinal cord PCTV, and iso3 was for the lower spinal cord PCTV. The field set iso1 consisted of five or seven fields with an average gantry angle, and the field sets iso2 and iso3 consisted of fields with three angles: 240°, 120°, and 180°.

### 2.C Radiation therapy guided by the ExacTrac system

**Fig. 1:**
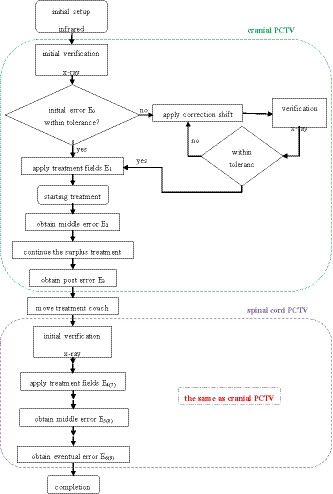
Flowchart of the workflow from setup to treatment completion using the ExacTrac system.

The ExacTrac system is based on a Varian Trilogy linear accelerator (Varian Medical Systems), which adopts 6‐MV photon and a sliding‐window technique. The accelerator is equipped with a high‐definition multileaf collimator (HD120 MLC) containing 120 leaves, 64 2.5 mm central leaves, and 56 5.0 mm peripheral leaves. The ExacTrac system was used for positioning in six directions (Lat (lateral), Lng (longitudinal), Vrt (vertical), Pitch, Roll, and Yaw and this order was followed throughout the article) and the flowchart of the workflow from patient setup to treatment was shown in Fig. 1. Firstly, the patient was positioned on the 6DoF couch and the couch was in the zero position at this moment, which meant the pitch, roll, and yaw angle were all set to 0°. Afterward, the patient was prepositioned with the aid of the infrared system, and this step was referred to as an initial setup that was then verified by using the x‐ray component of ExacTrac and matched to the corresponding reference digitally reconstructed radiograph (DRR) automatically by a corresponding matching algorithm. The verification of the initial patient setup was referred to as initial verification and the setup error resulting from this initial verification was referred to as initial setup error (E_0_). If the initial setup error was outside the tolerance (±2mm, ±2°), the determined correction was applied with the aid of the 6DoF couch. Two‐kV images with ExacTrac had been acquired again until the setup error (E_1_) was within tolerance, and then it was applied to fields. The position error was acquired similarly for the midtreatment (“mid‐treatment” meant when half of all treatment fields were delivered) and posttreatment recorded as E_2_ and E_3_. In the next step, the couch was moved from the first treatment isocenter to the second. Once the couch was moved, the new patient position was verified with the aid of the x‐ray system of ExacTrac. Again, if the resulting moving couch error was outside the tolerance, the corresponding correction was applied and again verified until the error was within tolerance (E_4_). The position error was also acquired for the midtreatment and posttreatment recorded as E_5_ and E_6._ In the third step, the second procedure was repeated for the third segment target volume and recorded position error as E_7_, E_8_, E_9_.

E_0_ was used to calculate the initial setup error. The postcorrection ExacTrac scan E_1_ or precorrection scan E_0_, where the initial setup was within tolerance, was used to calculate the residual setup error. Nine/six ExacTrac scans (E_1_ to E_9_/E_1_ to E_6_) of three/two isocenters target volume were conducted to monitor the intrafraction error. The variability E_m_ of the transposition of isocenters was obtained, and the overall position variability on the junctions was obtained by summing the mean position variability between adjacent targets.

The displacement of patients was related to the duration of radiation delivery, ordinal number of fraction (named i), and time points of image acquiring (named t_j_). Therefore, r_ij_, defined with the displacement vector of the patient, would be obtained with Lat, Lng, Vrt, Pitch, Roll, Yaw_j_. The misalignment of associated beam between two consecutive images was estimated by the following equation: following equation:$\varepsilon_{\mathrm{ij}}=r_{i,j+1}-r_{i,j}$and the systematic error$\sum_{\text{i}}^{}$for the fraction i can be obtained by

(1) $$\sum_{i}=N_{f,i}^{{}^{-1}}\sum_{j=1}^{{}_{{}_{N_{f,i}}}}\varepsilon_{\mathrm{ij}},$$

while$N_{f,i}$was the total number of images acquired in the fraction i. The random error$\sigma_{\text{i}}^{}$for the fraction i also could be estimated from

(2) $$\sigma_{\text{i}}={\left[N_{f,i-1}^{-1}\sum_{j=1}^{N_{f,i}}{\left(\varepsilon_{\mathrm{ij}}-\sum_{i}^{}\right)}^{2}\right]}^{1/2}.$$

These values enabled us to obtain the mean population setup errorMas

(3) $$M=N_{\text{s}}^{-1}\sum_{i=1}^{N_{s}}(\sum_{i}^{})$$

and the population random errorσas

(4) $$\sigma ={\left[N_{\text{s}}^{-1}\sum_{i=1}^{N_{\text{s}}}\sigma_{i}^{2}\right]}^{1/2}.$$

Where$N_{\text{s}}$was the total number of fractions included in this study.

Finally, the population systematic error∑was defined as the standard deviation of the patient systematic error, as follows:

(5) $$\sum ={\left[{\left(N_{s}-1\right)}^{-1}\sum_{i=1}^{N_{s}}{〈\sum_{i}^{}-M〉}^{2}\right]}^{1/2}.$$

The mean population setup error *M*, the population random error σ, and the population systematic error Σ were calculated via Eqs. (3), (4), (5).^18^, ^19^, ^20^, ^21^ The overall values of Σ and σ were defined as the root‐mean‐square of the setup error, the intrafraction error, and the transposition error of the isocenters. The geometric formula 2 Σ±0.7σ that was defined by van Herk^19^ was used to calculate the margins of the CTVs to PCTVs.

### 2.D Dosimetric effects of the longitudinal direction

To simulate the dosimetric effects of residual positional uncertainties, the original plan was copied to each new plan and recalculated in TPS using the same beam configuration and same total monitor unit, with different simulated isocenter position. The roll and yaw were rotated by changing the gantry angles and couch angles, respectively, but the rotation of the pitch was not an easy task. Some investigators^22^, ^23^ suggested that the dosimetric influence of small rotational errors was minimal for most cases in head, neck, and spine treatment. To simplify the simulation, this study only considered the residual error in the longitudinal direction for the time being. About 16(10) additional plans were generated for three isocenters (two isocenters) on the planning computed tomography images,^24^ for a total of 204 plans. The new plans included single isocenter shift plans ± 1mm, ±2 mm, and two isocenters shift 1mm, 2mm toward each other in the longitudinal direction.^25^ Tumor volume coverage and the maximal dose to the spinal cord were compared with those of the original plan.

### 2.E Follow‐up

Follow‐up began upon completion of the patients’ radiotherapy. Craniocerebral MRI and spinal cord MRI examinations of patients were conducted every 3 months during the first 6 months and every 6 months after 6 months of radiotherapy. The tumor size, recurrence, metastasis were monitored and compared. Side effects were evaluated by RTOG acute radiation injury grading standard and CTCAE3.0 standard.

## 3 RESULTS

A total of 2682 ExacTrac images, namely, 678 precorrection images (263 for setup and 261 and 154 for two transpositions of the isocenters) and 2004 images during the treatment delivery (postcorrection, middle‐treatment, and posttreatment), were acquired. The first to sixth images were acquired from all 15 patients, and seventh to ninth images were acquired from nine patients with three isocenters.

### 3.A Daily setup error and transposition error of the isocenters

**Fig. 2:**
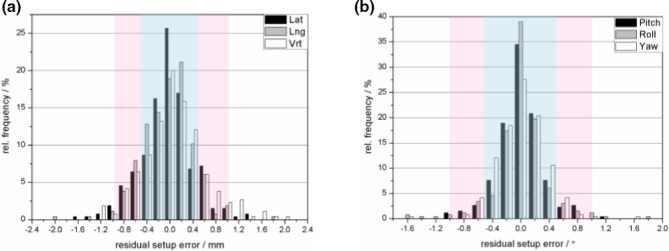
Histograms of the residual setup error. The areas in blue and pink corresponded to tolerances of (±0.5 mm, ±0.5°) and (±1.0 mm, ±1.0°). The data were binned into 0.2mm and 0.2° intervals. (a) Histogram for the translation direction. (b) Histogram for the rotation direction.

Data on the setup error from a total of 526 ExacTrac images from 15 patients are summarized in Table II, and the histograms showing the residual setup errors are plotted in Fig. 2. The postcorrection errors were within the tolerance and were smaller than the precorrection errors for all treatment fractions in the six directions. For the initial setup error, larger deviations occurred in the Lng and Roll directions. The attainment rates within the tolerance (±2 mm, ±2°) in the six directions were 76.81%, 66.79%, 76.81%, 88.59%, 76.72%, and 92.4%. The mean ± SD values were (−0.5 ± 1.8) mm, (0.4 ± 2.1) mm, (0 ± 1.7) mm, (−0.3 ± 1.3)°, (0.4 ± 1.8)°, (0 ± 1.2)°. Only three patients were within tolerance in the translation direction, and seven patients in the rotation direction for all fractions.

**Table II:** Overview of mean, minimum (min), maximum (max), and standard deviation (SD) value of the daily setup error and transposition error of isocenters in the six directions.

				Lat[mm]	Lng[mm]	Vrt[mm]	Pitch[°]	Roll[°]	Yaw[°]
Daily setup error	Initial		mean	−0.5	0.4	0.0	−0.3	0.4	0.0
			Min	−5.3	−6.7	−5.2	−6.7	−5.4	−4.6
			Max	4.5	5.7	4.9	3.5	6.9	4.0
			SD	1.8	2.1	1.7	1.3	1.8	1.2
	Residual		mean	0.0	0.0	0.1	0.0	0.0	0.0
			Min	−1.6	−1.9	−1.3	−1.0	−1.6	−1.5
			Max	1.5	1.9	2.0	1.2	1.8	1.7
			SD	0.5	0.4	0.5	0.3	0.4	0.3
Transposition error of centers	Initial	second PCTV	mean	−0.7	2.0	1.1	−0.1	0.2	−0.5
			Min	−14.7	−5.6	−11.9	−5.1	−2.4	−4.7
			Max	9.5	15.9	10.2	2.2	2.7	3.0
			SD	4.5	4.0	3.1	1.0	0.9	1.4
		third PCTV	mean	−0.5	2.5	−0.3	0.5	−0.4	−0.5
			Min	−24.8	−5.9	−7.6	−3.3	−5.9	−4.9
			Max	15.5	11.9	6.6	2.4	1.9	3.4
			SD	7.1	3.6	3.0	1.0	1.7	1.8
	Residual	second PCTV	mean	0.1	−0.1	0.0	0.0	0.0	−0.1
			min	−1.7	−2.3	−1.5	−1.6	−1.9	−1.1
			max	2.0	1.3	1.3	0.9	1.2	0.9
			SD	0.6	0.5	0.4	0.3	0.4	0.3
		third PCTV	mean	0.0	−0.2	0.1	0.0	0.1	0.0
			min	−2.0	−1.3	−1.2	−0.6	−0.8	−0.9
			max	1.6	0.6	1.5	0.8	0.8	0.8
			SD	0.8	0.4	0.5	0.2	0.3	0.3

The areas in blue and pink (Fig. 2) were visualizing the range where the residual setup error was within tolerance (±0.5 mm, ±0.5°) and (±1 mm, ±1°). The corresponding attainment rates were 81.51%, 83.40%, 75.85%, 91.67%, 89.77%, 93.21% and 95.47%, 97.74%, 91.70%, 99.62%, 97.35%, 99.25%, respectively. The mean ± SD values of the residual setup error were (0 ± 0.5) mm, (0 ± 0.4) mm, (0.1 ± 0.5) mm, (0 ± 0.3)°, (0 ± 0.4)°, (0 ± 0.3)°.

Data on the initial and residual errors of the spinal cord PCTVs (the second and third PCTVs) due to the transposition of the treatment isocenters are also summarized in Table II. The distributions of the initial errors were almost within ± 16 mm (SD 4.5 mm) in the direction of translation and ± 6° (SD 1.3°) in the rotation direction except for one fraction that the minimum value of the error of third PCTV in the Lat direction is −24.8 mm, while the corresponding residual errors were within ± 2 mm and ± 1.25°, respectively. The most significant error was detected in the Lat direction.

The Dispersal of the initial errors were more substantial in the direction of translation than in the rotation direction, and the third PCTV was more significant than the second PCTV in six directions. The dispersion was more extensive in the Lat directions than the Lng and Vrt direction, especially for the third PCTV, the main reason was that the head–neck–shoulder thermoplastic mask could not cover part of the chest and all of the abdomen and made it easier for the patient to move laterally. The attainment rates of errors that were caused by shifting isocenters being within the specified tolerance (±2 mm) in the translation direction for the second PCTV were 42.94%, 46.89%, and 68.93% of the fractions, compared to 21.15%, 44.23%, and 53.85% for the third PCTV. For the direction of rotation, the initial errors were more dispersive in the Yaw direction compared to the Pitch and Roll directions. The attainment rates exceeded more than 90% of the fractions within the tolerance (±2°) for the two spinal cord PCTVs, except in the Yaw direction, where the rates were 86.44% for the second PCTV and 71.15% for the third PCTV.

For the residual error, the attainment rates of the second PCTV were 65.91%, 72.73%, 83.52%, 90.91%, 88.07%, and 86.36% within (±0.5 mm, ±0.5°) and 90.34%, 94.89%, 96.02%, 99.43% 97.73, and 99.43% within (±1.0 mm, ±1.0°) in the six directions. The attainment rates of the third PCTV were 59.22%, 74.76%, 73.79%, 97.09%, 88.35%, and 93.20% within (±0.5 mm, ±0.5°) and 82.52%, 98.06%, 94.17%, 100%, 100%, and 100% within (±1.0 mm, ±1.0°).

We conclude that the position must be corrected after the daily setup and the transposition of the isocenters.

### 3.B Intrafraction motion

The delivery of treatment took 21.81 min (SD, 3.92 min) for the three isocenters plan. Ignoring the preparation time, the mean time intervals between scans were 3.37 min (SD, 0.80 min) for cranial PCTV and 1.44 min (SD, 0.17 min) for spinal cord PCTV.

According to 2004 images from nine scans, the intrafraction variability is summarized in Table III. The Dispersal of the data was found larger in the translation direction than in the rotation direction. The position was corrected at the first, fourth, and seventh scans by considering the setup and transposition of the isocenters. The position deviation was lowest after correction, and the SD satisfied first < second< third, fourth < fifth< sixth, and seventh < eighth< ninth in the six directions. The fifth, sixth, eighth, and ninth scans showed larger offsetting in the Lat direction compared to the other directions and fractions.

**Table III:** The value of the mean, min, max, and standard deviation of the intrafraction motion for nine scans in the six directions.

	Lat[mm]				Lng[mm]				Vrt[mm]			
	mean	min	max	SD	mean	min	max	SD	mean	min	max	SD
1^st^	0.0	−1.9	1.5	0.5	0.0	−1.4	1.9	0.4	0.1	−1.3	2.0	0.6
2^nd^	−0.1	−2.1	2.4	0.6	0.0	−1.3	1.3	0.5	0.0	−3.0	2.0	0.6
3^rd^	−0.1	−1.8	2.7	0.6	0.0	−2.9	1.9	0.5	0.0	−3.3	2.5	0.7
4^th^	0.1	−1.7	2.0	0.6	−0.1	−1.9	1.9	0.6	0.0	−1.7	1.3	0.4
5^th^	0.2	−2.9	4.0	0.9	−0.1	−2.3	1.8	0.6	0.0	−1.6	2.6	0.5
6^th^	0.1	−3.0	4.3	0.9	−0.1	−1.8	1.8	0.6	0.0	−1.6	2.7	0.5
7^th^	0.1	−2.0	1.6	0.7	−0.2	−1.3	0.8	0.4	0.1	−1.2	3.7	0.5
8^th^	0.2	−2.5	2.5	0.9	−0.2	−1.9	2.1	0.5	0.1	−1.3	2.3	0.5
9^th^	0.1	−4.0	2.2	1.0	−0.3	−1.9	1.8	0.5	0.1	−1.7	2.6	0.6

	Pitch[°]				Roll[°]				Yaw[°]			
	mean	min	max	SD	mean	min	max	SD	mean	min	max	SD
1^st^	0.0	−1.0	1.2	0.3	0.0	−1.6	2.8	0.4	0.0	−1.5	1.7	0.3
2^nd^	0.0	−2.5	1.5	0.4	0.0	−1.5	2.5	0.5	−0.1	−1.1	1.0	0.4
3^rd^	0.0	−1.8	2.8	0.5	0.0	−1.6	2.8	0.5	0.0	−1.0	1.2	0.4
4^th^	0.0	−1.6	1.1	0.3	0.0	−2.9	1.2	0.4	0.0	−1.1	1.0	0.3
5^th^	0.0	−2.5	1.7	0.4	0.0	−1.3	2.0	0.4	0.0	−1.3	1.3	0.4
6^th^	0.0	−2.1	3.4	0.4	0.1	−0.8	2.3	0.4	0.0	−1.2	3.2	0.4
7^th^	0.0	−0.6	0.8	0.2	0.0	−0.9	1.0	0.3	0.0	−0.9	0.9	0.3
8^th^	0.0	−1.1	0.5	0.3	0.0	−0.8	2.0	0.4	0.0	−1.0	1.3	0.3
9^th^	0.0	−1.0	1.4	0.3	0.0	−1.0	1.5	0.4	0.0	−1.0	1.2	0.3

**Fig. 3:**
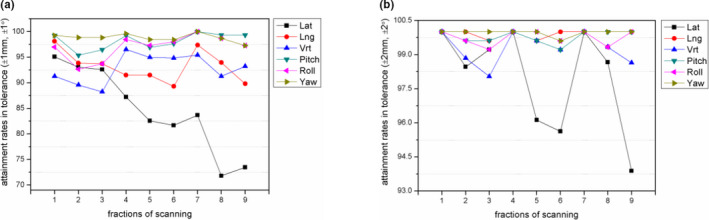
The attainment rates for the intrafraction motion in the six directions. (a) According to tolerance (±1mm, ±1°). (b) According to tolerance (±2mm, ±2°).

The attainment rates for tolerance (±1 mm, ±1°) and (±2 mm, ±2°) are plotted in Fig. 3. It could be seen that the attainment rates decreased gradually from the first scan to the ninth in the Lat direction, the last two scans accounted for 71.81% and 73.47% within (±1 mm, ±1°), and approximately 4%, 4.4%, and 6% of fractions had errors that exceeded 2 mm in the fifth, sixth, and ninth scans. The remaining five directions deteriorated according to similar trends; however, all results were almost within the tolerance.

Therefore, the intrafraction motion in the lateral direction should be monitored throughout treatment in consideration of the immobilization approach used in our department.

### 3.C Position variability in the junction areas

The data in Table IV reveal that the mean, min, max, and standard deviation value of the position variability for the two junctions in the six directions.

**Table IV:** The value of the mean, min, max, and standard deviation of the position variability for the two junctions in the six directions

	First junction						Second junction					
	Lat	Lng	Vrt	Pitch	Roll	Yaw	Lat	Lng	Vrt	Pitch	Roll	Yaw
mean	−0.2	0.2	0.0	0.0	0.0	0.0	−0.1	0.2	−0.2	0.0	0.0	−0.1
min	−2.9	−1.1	−2.4	−1.1	−1.7	−1.3	−2.1	−1.6	−2.5	−1.5	−1.5	−1.1
max	2.1	2.1	1.8	1.7	2.5	2.0	3.6	1.9	1.3	1.2	1.0	0.9
SD	0.9	0.7	0.7	0.5	0.5	0.5	1.0	0.7	0.7	0.4	0.4	0.4

The numbers of fractions of the two junctions (first and ^second^ junction) were 176 and 105 for 15 patients. The mean position variability of the first junction in the six directions were (−0.2 ± 0.9) mm, (0.2 ± 0.7) mm, (0 ± 0.7) mm, (0 ± 0.5)°, (0 ± 0.5)°, and (0 ± 0.5)°. The corresponding values were (−0.1 ± 1.0) mm, (0.2 ± 0.7) mm, (−0.2 ± 0.7) mm, (0 ± 0.4)°, (0 ± 0.4)°, and (0 ± 0.4)° for the second junction area. The attainment rates of the first junction within (±2 mm, ±2°) were 95.45%, 98.86%, 99.43%, 100%, 99.43%, and 99.43% and those of the second junction were 97.14%, 100%, 98.10%, 100%, 100%, and 100% in the six directions.

Therefore, we can conclude the position errors in the junction areas are almost within the tolerance after correction.

### 3.D Data analysis

The mean (M), systematic (Σ), and random (σ) components for the daily setup errors, intrafraction errors, and transposition errors of the isocenter(s) are listed in Table V. The PCTV was divided into the cranial PCTV (the first PCTV) and the spinal cord PCTV (the second PCTV) or into the cranial PCTV (the first PCTV), the upper spinal cord PCTV (the second PCTV), and the lower spinal cord PTV (the third PCTV). The setup error was divided into initial and residual setup errors, and the transposition error of the isocenter(s) was split into initial and residual errors of the second PCTV (third PCTV).

**Table V:** Overview of mean, systematic, and random components of the setup error, intrafraction error, and transposition error of isocenters in six directions

			Lat[mm]	Lng[mm]	Vrt[mm]	Pitch[°]	Roll[°]	Yaw[°]
Daily setup error	Initial	M	−0.5	0.4	0	−0.3	0.4	0
		Σ	1.4	1.7	1.5	1.0	1.3	0.9
		σ	1.2	1.3	1.0	0.9	1.3	0.9
	Residual	M	0	0	0.1	0	0	0
		Σ	0.1	0.2	0.2	0.1	0.2	0.1
		σ	0.5	0.4	0.5	0.3	0.4	0.3
Intrafraction error		M	0	0	0	0	0	0
		Σ	0.1	0.1	0.1	0.1	0.1	0.1
		σ	0.7	0.7	0.6	0.4	0.4	0.4
Transposition error of isocenters	second PCTV before correction	M	−0.6	1.2	1.6	0.1	0.3	−0.4
		Σ	3.1	3.0	2.6	0.7	0.5	0.8
		σ	3.4	2.6	2.1	0.7	0.8	1.1
	third PCTV before correction	M	−0.2	2.1	0.2	0.4	−0.1	−0.1
		Σ	5.8	1.9	1.6	0.4	1.1	1.3
		σ	5.0	3.2	2.4	0.8	1.0	1.5
	second PCTV after correction	M	0.1	−0.1	0	0	0	0
		Σ	0.3	0.3	0.2	0.1	0.1	0.1
		σ	0.8	0.7	0.7	0.3	0.4	0.3
	third PCTV after correction	M	0.1	−0.2	0.1	0	0	0
		Σ	0.4	0.2	0.2	0.3	0.1	0.1
		σ	0.6	0.4	0.5	0.2	0.3	0.3

The values of Σ and σ of the residual setup error were significantly lower than those of the initial setup error. The systematic and random components of the initial setup error were both close to or higher than (±1 mm, ±1°). The mean elements of the residual setup errors were lower than 0.1 mm in the translation direction and 0.01° in the rotation direction. The random error was more significant than the systematic error. For intrafraction motion, the random component was more significant than the systematic part in all six directions, and the mean component was less than 0.03 mm in the translation direction and 0.005° in the rotation direction, which were both lower than the setup error. The systematic part was smaller than the residual setup error, except in the Lat direction (0.13 mm). For the second and third PCTVs, the systematic and random elements of the initial error were abundant in the translation direction, for example, Σ = 3.09 mm and σ = 3.36 mm for the second PCTV and Σ = 5.79 mm and σ = 5.03 mm for the third PCTV in the Lat direction. The mean component was lower than 0.1 mm and 0.3° for the second PCTV and 0.2 mm and 0.2° for the third PCTV. The systematic and random parts of the residual error were far less than the initial error. The systematic and random components of the residual error were also more substantial in the Lat direction than in the other five directions.

Therefore, if no daily image is monitored, the CTV‐to‐PCTV margin is (13 mm, 11 mm, 10 mm, 5°, 6°, 6°), while if controlled by ExacTrac, the margin is (2 mm, 2 mm, 2 mm, 2°, 1°, 1°) that is in the range of target expansion criteria.

### 3.E Dosimetric effects of the longitudinal direction

Statistical results of normalized dose differences of PCTV, PGTV, and spinal cord between recalculated doses and original doses accounting for the longitudinal shift are listed in Table VI. The new plans were regained, including isocenters shift −1 mm, −2 mm, 1 mm, 2 mm individually, and adjacent isocenters moved toward each other at about 1 mm, 2 mm. These isocenter shift modes are referred to as error category.

**Table VI:** The normalized dose differences of PCTV, PGTV, and spinal cord between recalculated doses and original doses.

Error category	PCTV																		
	Min dose					Max dose					Mean dose					HI			
	mean	min	max	SD		mean	min	max	SD		mean	min	max	SD		mean	min	max	SD
F−0.1	1.03	1.00	1.19	0.05		1.00	1.00	1.02	0.01		1.00	1.00	1.00	0.00		0.76	0.01	1.03	0.44
F−0.2	1.06	0.95	1.25	0.09		1.01	1.00	1.05	0.02		1.00	1.00	1.01	0.00		0.77	0.02	1.05	0.44
F + 0.1	0.94	0.80	1.00	0.07		1.00	0.99	1.00	0.00		1.00	1.00	1.00	0.00		0.75	0.00	1.00	0.43
F + 0.2	0.89	0.60	1.00	0.14		1.01	1.00	1.09	0.02		1.00	0.99	1.00	0.00		0.68	0.00	1.01	0.43
S−0.1	1.02	0.99	1.18	0.05		1.00	0.99	1.01	0.00		1.00	1.00	1.00	0.00		0.76	0.00	1.03	0.45
S−0.2	1.02	0.85	1.28	0.10		1.01	0.99	1.09	0.02		1.00	0.99	1.00	0.00		0.76	0.01	1.06	0.43
S + 0.1	0.98	0.81	1.00	0.05		1.00	0.99	1.01	0.01		1.00	1.00	1.00	0.00		0.76	0.00	1.02	0.44
S + 0.2	0.97	0.67	1.00	0.09		1.01	0.99	1.03	0.01		1.00	1.00	1.01	0.00		0.76	0.01	1.03	0.44
T−0.1	0.90	0.04	1.07	0.33		1.01	1.00	1.10	0.03		1.00	0.98	1.00	0.01		0.78	0.00	1.00	0.41
T−0.2	0.91	0.04	1.16	0.33		1.01	1.00	1.10	0.03		1.00	0.98	1.00	0.01		0.78	0.00	1.00	0.41
T + 0.1	0.88	0.03	1.00	0.32		1.01	1.00	1.11	0.04		1.00	0.97	1.00	0.01		0.78	0.00	1.00	0.41
T + 0.2	0.86	0.03	1.00	0.32		1.01	1.00	1.11	0.04		1.00	0.97	1.00	0.01		0.78	0.00	1.00	0.41
F−0.1S + 0.1	0.99	0.04	1.18	0.27		1.02	0.99	1.10	0.03		1.00	0.98	1.01	0.01		0.78	0.02	1.06	0.41
F−0.2S + 0.2	1.02	0.04	1.28	0.29		1.05	1.00	1.15	0.05		1.00	0.98	1.01	0.01		0.79	0.04	1.12	0.41
S−0.1T + 0.1	0.88	0.03	1.01	0.32		1.01	1.00	1.11	0.04		1.00	0.97	1.00	0.01		0.78	0.00	1.00	0.40
S−0.2T + 0.2	0.87	0.03	1.01	0.32		1.02	1.00	1.11	0.04		1.00	0.97	1.00	0.01		0.78	0.00	1.00	0.40

	PGTV												Spinal cord			
	Min dose				Max dose				Mean dose				Max dose			
	mean	min	max	SD	mean	min	max	SD	mean	min	max	SD	mean	min	max	SD
F−0.1	1.01	0.99	1.03	0.01	1.00	0.99	1.02	0.01	1.00	1.00	1.01	0.00	1.03	1.00	1.15	0.04
F−0.2	1.00	0.98	1.05	0.02	1.01	0.98	1.05	0.02	1.00	1.00	1.01	0.00	1.08	1.02	1.21	0.06
F + 0.1	0.98	0.93	1.00	0.02	1.00	0.99	1.00	0.00	1.00	0.99	1.00	0.00	1.01	0.97	1.24	0.06
F + 0.2	0.96	0.85	1.01	0.04	0.93	0.00	1.01	0.26	1.00	0.99	1.00	0.00	1.01	0.99	1.24	0.06
S−0.1	1.00	0.98	1.03	0.01	1.00	1.00	1.01	0.00	1.00	0.99	1.00	0.00	1.00	0.95	1.01	0.01
S−0.2	0.99	0.91	1.06	0.03	1.00	0.99	1.03	0.01	1.00	0.99	1.01	0.00	1.02	0.99	1.07	0.03
S + 0.1	1.00	0.98	1.02	0.01	1.00	1.00	1.01	0.00	1.00	1.00	1.00	0.00	1.02	1.00	1.08	0.02
S + 0.2	1.00	0.94	1.03	0.02	1.01	1.00	1.03	0.01	1.00	1.00	1.01	0.00	1.08	1.04	1.22	0.05
T−0.1	1.00	1.00	1.00	0.00	1.00	1.00	1.00	0.00	1.00	1.00	1.00	0.00	1.04	0.99	1.37	0.12
T−0.2	1.00	1.00	1.00	0.00	1.00	1.00	1.00	0.00	1.00	1.00	1.00	0.00	1.04	0.99	1.37	0.12
T + 0.1	1.00	1.00	1.00	0.00	1.00	1.00	1.00	0.00	1.00	1.00	1.00	0.00	1.05	1.00	1.37	0.12
T + 0.2	1.00	1.00	1.00	0.00	1.00	1.00	1.00	0.00	1.00	1.00	1.00	0.00	1.05	0.99	1.38	0.12
F−0.1S + 0.1	1.00	0.93	1.04	0.03	1.01	0.99	1.06	0.02	1.00	1.00	1.01	0.00	1.11	1.03	1.37	0.09
F−0.2S + 0.2	1.00	0.91	1.09	0.04	1.02	0.98	1.12	0.04	1.00	0.99	1.02	0.01	1.19	1.09	1.37	0.09
S−0.1T + 0.1	1.00	0.98	1.00	0.01	1.00	0.99	1.00	0.00	1.00	0.99	1.00	0.00	1.08	1.00	1.38	0.13
S−0.2T + 0.2	0.99	0.96	1.00	0.01	1.00	0.99	1.00	0.00	1.00	0.99	1.00	0.00	1.13	1.04	1.38	0.12

Abbreviations: F = the first isocenter, S = the second isocenter, T = the third isocenter.

+: the introduced errors were in the same direction to the coordinate axis;

−: the introduced errors were in the opposite direction to the coordinate axis;

The max dose of PGTV had a maximum increase of 12.1%, the minimum dose had a maximum decrease of 15.3%, and the mean doses fluctuated within 2.1%. PGTV was not affected by the introduction errors of the third target. Errors of 1 mm and 2 mm in a single target and of 1 mm and 2 mm in two head‐on targets decreased the min doses by 0.23%, 0.97%, 0.16%, and 0.62% on average and increased the max doses by 0.11%, −0.85%, 0.46%, and 0.93% on average, while the mean doses decreased by 0.009%, 0.036%, −0.006%, and 0.043% on average.

The max dose of PCTV showed a maximum increase of 15%, and the minimum dose showed a maximum decrease of 39.7%, except for patient 5, for whom the decline was 96.7%, and the mean doses fluctuated within 0.027%. Errors of 1 mm and 2 mm in a single target and 1 mm and 2 mm in two head‐on targets decreased the min doses by 4.01%, 5.02%, 6.41%, and 5.58% on average and increased the max doses by 0.47%, 0.92%, 1.60%, and 3.66% on average, while the mean doses decreased by 0.090%, 0.102%, 0.206%, and 0.216% on average.

In considering the max dose of the spinal cord, the introduced errors resulted in 67.5% fractions exceeding the original value. Errors of 1 mm and 2 mm in a single target increased the dose by 2.4% and 4.7% on average, and errors of 1 mm and 2 mm in two head‐on targets increased the dose by 9.9% and 16.1% on average. These results were within the scope of normalization of 1.375. Therefore, the max dose limit of the spinal cord must be less than 3273 cGy in the clinic routine for security.

In terms of HI of PGTV and PCTV, 60.2% and 58.8% of fractions increased, 0.5% and 2.9% remained unchanged, and 39.3% and 38.2% decreased. The HI of PGTV showed a maximum increase of 108.7%, compared to 56.6% for PCTV, except for patient 5, for whom the maximum growth was 115.7%.

### 3.F Follow‐up

The study was continued until May 2019, and the median follow‐up period was 20.3 (15–30) months. Fifteen patients survived with no local or distant recurrences of tumor or spread of spinal cord. One patient developed cerebral edema 17 months after radiotherapy, and the extent of cerebral edema decreased after treatment. Radiation myelitis was not observed after the craniospinal radiotherapy.

Therefore, the patients did not exhibit any side‐effects by the overall treatment during the follow‐up period.

## 4 DISCUSSION

The ExacTrac image‐guided radiotherapy system provides a fast and effective method for monitoring the position of the patients receiving craniospinal irradiation, and the cumulative dose is about 0.5–1 cGy in this study.

In this study, the initial setup errors in more than 96% of the fractions were in the range of (±4 mm, ±4°) in six directions according to the result of Beltran,^11^ namely, children who were not localized using CBCT had a setup uncertainty of as large as 4 mm. According to Beltran, errors > 2° should be corrected due to the nonnegligible changes in the gEUD for critical structures or target volumes. Peng et al.^26^ demonstrate that rotational setup errors < 3° have a very minimal influence on the dose distribution.

Physiological movements such as respiratory motion, peristaltic motion, and heartbeat increase the setup uncertainty^27^ and may result in deviation of the irradiated volume and organs at risk.^28^, ^29^ The treatment times for CSI delivery are often approximately 20 min or longer, which is associated with a higher risk of positional variation.^30^ In our study, the delivery also took at least 20 min for a three‐isocenter plan. Iglesias et al.^31^ adopt three isocenters, and two arcs are planned for each isocenter, which are image‐guided with CBCT. The total treatment time, including image acquisition, was approximately 30 min. Theoretically, the magnitude and probability of intrafraction variability will most likely increase with the delivery time. Hoogeman^30^ and Thierry^32^ conclude that the intrafraction systematic error increases with time, as patients drift away from their initial setup position during treatment. Repeating image acquisition and patient positional correction at an interval of less than 5 min may sufficiently reduce the error that is associated with intrafraction patient motion. Willoughby^33^ and Hoogeman et al. have investigated the frequent acquisition of images every 0.5–2 min and continuous, real‐time tracking of the target volume during radiation delivery with technologies such as CyberKnife and electromagnetic localization.^33^, ^34^ In our study, the images acquisition frequencies were 4.1 min for a cranial target volume and 1.45 min for a spinal cord target volume. However, patients may move unconsciously during radiation delivery, and differences in the measurement and acquisition schedules may lead to discrepancies between the measured and actual motions. Therefore, continuous imaging should be performed to evaluate the intrafraction action. Immobilization devices can be regarded as another significant factor. The intrafraction systematic error in our study 0.08–0.13 mm is lower than 0.3–0.7 mm during an approximately 15‐min IMRT delivery.^35^

Transposition of treatment isocenter(s) is a significant factor that affects the hot and cold dosage spots in junction regions. Seppala et al.^10^ propose a dynamic sfIMRT technique in CSI with isocenters that overlap at least 4 cm with each other, and the main benefit is that the homogeneous dose distribution is insensitive to alignment errors. Fred et al.^9^ propose a similar technique that uses a three‐isocenter jagged‐junction IMRT plan that involves the realization of overlap in the junction regions using multiple beam directions: three for the spinal junctions and ten for the CSI junction. According to Fred, that their approach reduces the susceptibility of the junction areas to mismatching and the formation of hot and cold spots, as an error in one beam direction will not be compounded but will be compensated for by other beams. We ensure that each target volume remains in the same position as planned throughout the treatment via the guidance of the ExacTrac system to avoid hot spots and cold spots of dosage. Paolo et al.^36^ adopt a combination of two couch techniques, one after the other, for feathering. The helical tomotherapy^37^, ^38^ technology delivers the entire treatment without junctions, thereby reducing the risk of geometric error and improving HI and CI. However, the associated low‐dose radiation “bath” increases the risk of secondary malignancy and other late severe effects.^39^

The introduced errors resulted in a 96.7% reduction in the min dose of the spinal cord target volume in one patient, although the mean doses fluctuated within 0.027%; hence, a small portion of the target volume was not exposed. Inhomogeneity of the dose due to position errors can result in under dosage and local relapses in the cribriform and neuraxis areas.^40^, ^41^ Paulino et al. measure the dose that is delivered to the craniospinal using the ExacTrac image system and verify the result by analyzing radiochromic film that is acquired in a 30 × 30 × 10 cm^3^ solid water phantom and by comparison with verification plans. The dose difference in the junction region is at most 8%. Seppala et al. report the mean max and min doses in the spinal cord PCTV are 107.0% and 91.7%, respectively, of the prescribed dosage. In this study, the max and min doses of PCTV are 115% and 3.3% of the prescription dose, and the mean doses fluctuate within 0.027%. Thus, there are junction areas that do not receive the full prescribed dose of radiation, and the mean dose is not significantly affected.

Our study had some potential limitations. First, the dosimetric effects of longitudinal direction were only considered; the other directions will be simulated in the subsequent research. The roll, yaw, and pitch will be simulated by changing the gantry angles, couch angles, and rotating the planning CT image around the isocenter, respectively. Second, the sample was small, and the follow‐up was not long enough, the conclusion could be supported by increasing the number of samples and continue to follow‐up. Third, the ExacTrac system relies on accurate bony anatomy alignment for patient positioning. However, target deviation inside the skull attributable to the alleviation of perilesional edema by concomitant antiedema treatment or geometrical change of the target owing to early tumor shrinkage may occur during or even before treatment. Precautions, therefore, need to be taken against these possibilities with careful neurological monitoring or neuroimaging evaluation if needed.^42^

## 5 CONCLUSIONS

The ExacTrac is an effective system for evaluating and improving the accuracy of sfIMRT in CSI patients. Position correction is necessary after setup and transposition of the treatment isocenter(s). Intrafraction motion in the lateral direction should be monitored throughout treatment. The mean position variability in the junction areas was almost within the tolerance (±2 mm, ±2°) after correction by the ExacTrac system and the residual error had little effect on the mean dose of the target volume. In contrast, the min dose and max dose of the target volumes and the max dose of the spinal cord had to be constantly monitored. The patients did not exhibit any side effects by the overall treatment during the follow‐up period.

## CONFLICT OF INTEREST

The authors have no conflicts to disclose.
